# What Some of the Preachers Said

**Published:** 1893-06-17

**Authors:** 


### The Institutional Workshop.

### HOSPITAL SUNDAY.

### WHAT SOME OF THE PREACHERS SAID.

St. Paul's Cathedral.

The morning service at St. Paul's Cathedral was attended in state by Alderman Sir James Whitehead, M.P. (acting for the Lord Mayor), who was accompanied by Mr. Alderman and Sheriff Wilkin and Mr. Under-Sheriff Rose-Innes. The sermon was preached by the Rev. Prebendary Tucker from St. Luke x. 30, "Take care of him." He said that he did not base for a Christian congregation the duty of caring for their sick and suffering brethren on the consideration of common humanity or philanthropy. Such an argument was legitimate in its own place, but that place was not within those walls. Many would, he knew, contribute that day to the maintenance of the hospitals of London who never bowed the knee at the Christian altar, nor worshipped in the congregation of the faithful, and might God bless them for their benevolent act. But to those who were worshipping that day in that cathedral church he rested the duty on loftier arguments. It was none other than the Lord of compassion and goodness who said to them, "Take care of all who need your help." There was a sense in which the members of the Christian Church would always have all things in common, for God, who weaved, by His providence, the threads of all men's lives into one common woof, brought one man's fulness into relation with another man's lack, and provided, if men would fulfil their parts, that no member of the spiritual body should want. And this was a lesson which they in that city and in these times had to learn. They were apt to boast themselves of the multitude of their charities. Multitudinous, indeed, they were, crossing each other's paths, crowding each other out, worked at needless cost, and often appealing for support on unworthy and partisan grounds, until, by their incessant appeals, they wearied and repelled those who were not sorry to have the excuse of their distracting numbers to give to none of them. That was a matter of evil and unstatesmanlike administration, which common sense and repression of self might obviate. But when they turned from the needless array of these multitudinous organisations, which gave no grounds for boasting, and considered that every one of them was in a chronic state of want, that they were nearly all maintained by the very questionable means of bazaars, charity dinners, &c., and then turned to the simple words of the text, "Take care of him," and remembered Who it was that had laid this injunction on them, then their exultation must yield to self-abasement. If Christian people would revert to the system, which dated at least from the time of the Father of the Faithful, and sternly set apart the first tenth of their incomes to pious and charitable purposes, there would be money enough to meet every need.

Westminster Abbey.

There were three services at the Abbey, the congregations being very large. At the morning service Canon Teignmouth Shore was the preacher; Archdeacon Farrar conducted the afternoon service, and Canon Scott Holland that held in the evening. The Lord Mayor was represented at the afternoon service by Sir James Whitehead, M.P., Lady Whitehead also being present, as were also the American Ambassador and Mrs. Bayard, the Bishop of Derry, the Dean of Hereford, and Sir R. Peel. Archdeacon Farrar, preaching from Psalm cxix. 68—"Thou art good and doest good; teach me Thy statutes"—said the Sunday which they had come to call "Hospital Sunday" was the festival of mercy; it was an opportunity to earn the beatitude of beneficence; it was the appeal for the compassion due to suffering humanity. Millions were living, though they tried to conceal it from themselves, like churlish Nabal, in sensual plenty. Let not Dives that day think that he sufficiently discharged his duty to the millions of Lazaruses at his gate because, out of thousands of pounds, he grudgingly spares to charity, as a grim duty, not as a happy privilege, a guinea here and there which he would not miss. It was the first axiom of a Christian's duty that no man liveth and no man dieth to himself. There was not one present who might not help to make the lazar-house of sickness, not "sad, noisome, dark," but cheerful with sunlight, bright with flowers, lit up with human faces which were a healing in themselves. Form stores of gold, selfishly squandered or meanly hoarded, they might send white-winged messengers of mercy to move amid the suffering multitude. Pain, disease, sickness, he proceeded, emphasised for them the eternal distinctions between right and wrong. Physical anguish and moral remorse, often in the individual, always for the race, were but a part of the stream of human sin, taken a little lower in its course. Of the 1,008 diseases to which the human frame was liable, whole groups were only other names for their own faults and vices—for impurity, for gluttonly, for the abuse of intoxicating drinks, for selfish greed about things earthly, for the fret and fuss of pushing competition and exorbitant desires. Man himself, if he would keep the Ten Commandments, if he would live in temperance, soberness, and chastity, might to an immense extent sweep his life clean of foul disease. Turning from the lessons which pain and sorrow have for the individual to that which they carried to society and the world in general, he did not hesitate to say they were the stern saviours of society as they were the stern missionaries to the individual soul.

Temple Church.

Dr. Vaughan, Master of the Temple, preached the morning sermon, taking for his text Psalm cxxxix. 13, "I will give thanks unto Thee, for I am fearfully and wonderfully made." He said that other thoughts lay in the text, but he would select two for special notice—first, the liability to injury implied in the "fearfully," and next, the provision for reparation implied in the "wonderfully." After tracing the two thoughts in their application to the threefold constitution of man's nature in body, mind, and soul, Dr. Vaughan ended his sermon with an earnest appeal for Hospital Sunday. He said: "Fearfully made" is the description of the human body. Fearfully made, and fearfully circumstanced; liable, every day, every hour, in each limb and each organ, to disease and danger, to pain and death. Such we are, such is life. Last year 98,000 patients were received into the hospitals of London; more than two millions and a half received treatment from them; 285,000 accidents and emergencies were ministered to by them. What a catalogue of personal suffering, of distressed, impoverished homes by reason of it, of silent appeals to human sympathy, to Christian charity, listened to or disregarded! Yet "wonderfully made" is the other half of the description. Upon these thousands, these hundreds of thousands, kindness, mercy, charity have tried their hands in relief and healing, and not in vain, under the impulse of humanity, under the skilled hand of science, under the compulsion of love. For 20 years now the institution of a Hospital Sunday has wrought its beneficent work. It has gathered from a steadily increasing number of Christian congregations more than £620,000 in aid of the infirmaries and dispensaries of London. And yet the highest amount hitherto collected on Hospital Sunday has provided but one-twentieth part of the annual expenditure of the hospitals, and the total sum of the 20 years' collections falls far, very far, below the cost of one year's hospital work. We cannot be proud of it.

The Great Synagogue.

The Chief Rabbi, pleading on Saturday at the Great Synagogue on behalf of the Hospital Sunday Fund, referred to the fact that the value of hospitals had been strikingly illustrated by two noteworthy instances that had recently occurred in his community. The pious and learned Rabbi Jacob Reinowitz, of whom he could say without exaggeration that he knew the whole Talmud by heart, whose demise they still deplored, had, when suffering from a grave malady, resorted to the London Hospital, and there had been treated with a consideration and tenderness beyond praise. Another veteran *savant*, Dr. Rabbinowicz, the able translator of the Talmud into French, had spent the last days of his life in the Westminster Hospital, where everything was done to relieve pain and to mitigate the mortal agony. It was then not without good reason that an infirmary, an asylum for the aged and sick, was termed, in the picturesque language of the Hebrews, a *Hekdesh*, a sanctuary, a shrine. The offertory amounted to about £260.

St. Peter's, Eaton Square.

The Rev. J. Storrs took for his text St. John xiii. 35, "By this shall all men know that ye are my disciples if ye have love to one another." The preacher spoke of the need of a practical test to show the value of our love for God. In what way could this be so well shown as by love for our neighbour? St. Peter places this first and highest—"Above all things have perfect charity." St. Paul says: "Greatest of these is charity." And the love which God commands and expects is not mere philanthropy, not merely love of mankind, but love of the individual. Humanity is, in the aggregate, often very repulsive, yet we have to believe that in every man there is some little measure of good. Mr. Stoors concluded his sermon by an appeal for the sick and suffering, asking all those present that morning to offer thanksgivings for the blessings they or their friends had received through that medical skill which was largely due to the existence of our hospitals. Love—this love of our neighbour would altogether have failed if there was no desire to give. He could only pray that these offerings would be prompted by love, and accepted by the love of God on High.

Holy Trinity, Sloane Square.

The Rev. R. Eyton, Holy Trinity, Sloane Square, after a discourse directed to point out the inconsistencies and imperfections to be expected in the best of religious lives, stated that he did not on this Sunday especially set apart for collections in aid of our hospitals, intend to make a sensational appeal. Such appeals by exciting spurious compassion and emotion frequently resulted in donations which were repented of as soon as the donors left the church. Nevertheless, he said he knew nothing better calculated to elevate the mind and remove impurities from our religious life than practical sympathy with others. In helping the poor and suffering around us we help ourselves, and what we cannot do ourselves we can be the means of assisting others to do for us, by contribution of that which we have.

St. George's, Bloomsbury.

At St. George's, Bloomsbury, Rev. A. B. Boyd Carpenter preached on St. Matthew viii, part of 16th verse, "And He cast out the spirits with His word, and healed all that were sick," and said that tending and healing the sick were parts of the work carried out consistently by Christ during His life upon earth; they were essentially a Christian's duties. The annual appeal for funds for the maintenance of hospitals and dispensaries was an established custom, and there was always the fear of custom becoming routine, and routine sinking into indifference. This ought not to be, and in one of the wealthiest cities of the world, where thousands of pounds were daily spent without stint for unnecessary luxuries, this terrible deficiency, this balance on the wrong side of hospital accounts was deplorable. Actually whole wards were closed for want of funds, not because they were not ready for use; everything was complete, the highest medical talent in the land, and the women with trained hands and willing hearts, all were available, but the generous supplies which should make these practically useful were, unfortunately, lacking. The present condition of our hospitals was a crying shame, which should indeed appeal to the national conscience. Such things ought not to be. Many arguments were put forward on the subject of hospitals; criticisms of public movements tended to keep them healthy, and it was a mistake for institutions to object to it. If "Hospital Sunday" can be proved to have lowered the total amount of subscriptions to the institutions, then assuredly it was a mistake. The plan was originally started to give those who could only offer small sums an opportunity of presenting their gifts. Doubtless those subscriptions which the publicity of a list helps to secure would dwindle down under the secrecy of the offertory bag, and unhappily one guinea might be substituted for the ten formerly given. This was no fault of Hospital Sunday, for these collections were intended to supplement, and not to withdraw, subscriptions. The preacher thought it was reasonable to expect and to hope that the gross receipts would be considerably augmented. If people made their offerings through the National Church to national charities they should be on the largest possible scale. Persons argued that to support hospitals was merely to "tinker up" a bad state of affairs, and that everybody should be paid well enough for their work to need no medical charity. It was so easy to say what "ought to be," but the great problem of poverty, the hardest we have to solve, will not be fathomed yet. Our dreams of Utopia will not be realised in the nineteenth century. We may go on hoping and trusting, but this will not excuse us from giving to-day. Following the example of Christ we must accept the work as we find it, and we must acknowledge existing facts of society and humanity. The reformations which may take place to-morrow are no reasons for neglecting the good we may do to-day. We must meet the immediate want whilst looking forward to the nobler future. Disease is present with us. Men, women, and little children are suffering and must be relieved. Existing evils have prepared them for hospitals, and we must help to lift the burden of disease from the sufferers. Talented men and noble women give their services and their lives to the sick, those who cannot do as much may at any rate give money and give it willingly and liberally. The great work taught us by Christ's example is to help those who are in need, whether of body or soul. He laid stress on the human body, He never said "this does not come within the range of my ministrations." Healthy, wholesome bodies must be ensured or there cannot be development of mind and spirit. In hospitals the poor are helped by the skill and the attention which they cannot get at home. There is no haphazard work in hospitals, the doctors leave no chance untried to restore health to those who are poor in pocket and in body too. Of few other things can we be so proud as of our hospitals. There the doctors wield a sword, not to destroy but to preserve lives by their skill, thought, and attendance. The rich are not justified in giving paltry sums because, as they say, they never need hospitals. But where did the skilled doctor whom they summon to their luxurious homes gain his knowledge save in the hospital? They await his visits with anxious eyes, but their gratitude for his science seldom extends to the institution where his skill and experience were acquired. As medical schools hospitals are of great value to rich and poor, young and old.

